# Top Questions in the Diagnosis and Treatment of Pulmonary *M. abscessus* Disease

**DOI:** 10.1093/ofid/ofz221

**Published:** 2019-05-14

**Authors:** Ruvandhi R Nathavitharana, Luke Strnad, Philip A Lederer, Maunank Shah, Rocio M Hurtado

**Affiliations:** 1 Division of Infectious Diseases, Department of Medicine, Beth Israel Deaconess Medical Center and Harvard Medical School, Boston, Massachusetts; 2 Division of Infectious Diseases, Department of Medicine, Oregon Health and Sciences University, Portland, Oregon; 3 Epidemiology Programs, Oregon Health and Sciences University and Portland State University School of Public Health, Portland, Oregon; 4 Section of Infectious Diseases, Department of Medicine, Boston Medical Center, Boston, Massachusetts; 5 Division of Infectious Diseases, Johns Hopkins School of Medicine, Baltimore, Maryland; 6 Baltimore City Health Department, Baltimore, Maryland; 7 Division of Infectious Diseases, Department of Medicine, Massachusetts General Hospital and Harvard Medical School, Boston, Massachusetts; 8 Global Health Committee, Ethiopia and Cambodia

**Keywords:** bronchiectasis, drug resistance, lung disease, mycobacterium abscessus, non-tuberculous mycobacteria

## Abstract

*Mycobacterium abscessus* disease is particularly challenging to treat, given the intrinsic drug resistance of this species and the limited data on which recommendations are based, resulting in a greater reliance on expert opinion. We address several commonly encountered questions and management considerations regarding pulmonary *Mycobacterium abscessus* disease, including the role of subspecies identification, diagnostic criteria for determining disease, interpretation of drug susceptibility test results, approach to therapy including the need for parenteral antibiotics and the role for new and repurposed drugs, and the use of adjunctive strategies such as airway clearance and surgical resection.

## Teaser

Mycobacterium abscessus pulmonary disease is particularly challenging to treat, given its intrinsic drug resistance and limited data to guide management. In this paper, we discuss commonly encountered clinical management questions including diagnostic criteria, interpretation of drug susceptibility testing, approach to treatment, including choice of intravenous versus oral antibiotics, and adjunctive strategies, including airway clearance and surgery.

*Mycobacterium abscessus* complex (*M. abscessus*) is part of a group of rapidly growing mycobacteria (RGM) that can be found in soil and water and accounts for the majority of pulmonary nontuberculous mycobacteria (NTM) infections due to RGM [1]. Given its increasing prevalence [2] and intrinsic multidrug resistance [3], to complement other review articles on this topic [4–7], this article will focus on common clinical questions that arise during the management of pulmonary *M. abscessus* disease.

## HOW DO I KNOW IF THE PATIENT HAS *M. ABSCESSUS* DISEASE WITH A SINGLE POSITIVE SPUTUM CULTURE?

In contrast to *M. tuberculosis*, a single culture from sputum or another nonsterile site that is positive for for *M. abscessus* does not necessarily indicate a person has *M. abscessus–*related disease. The American Thoracic Society/Infectious Diseases Society of America (ATS/IDSA) guidelines recommend assessment of clinical (including radiographic) and microbiological criteria to establish a diagnosis of pulmonary NTM disease [8]. Clinical criteria for pulmonary disease (warranting consideration of treatment) include pulmonary symptoms, nodular or cavitary opacities on chest radiograph, or multifocal bronchiectasis on chest computed tomography scan, along with appropriate exclusion of other diagnoses such as tuberculosis or malignancy. Microbiologic criteria comprise the identification of positive cultures from 2 separate sputum samples or 1 positive bronchial washing or lavage specimen (BAL), or pathologic features consistent with NTM disease in combination with a positive culture. As NTM, like *M. abscessus*, may be environmental organisms, care must be taken to distinguish between colonization, pseudo-outbreaks (eg, due to contaminated equipment such as bronchoscopes), lab or specimen contamination (eg, oral rinsing with contaminated tap water before specimen collection), and true disease [9, 10]. Therefore, the isolation of *M. abscessus* without associated clinical (including radiographic) features or a single positive sputum specimen, even with supporting clinical features, does not necessarily establish a diagnosis of pulmonary disease [3, 11]. Individuals with some pulmonary comorbidities, such as cystic fibrosis (CF), may be at increased risk for colonization, requiring additional consideration of other pathogens and clinical findings when determining the clinical significance of positive *M. abscessus* cultures [12]. For these reasons, fulfillment of microbiological criteria (per the ATS/IDSA guidelines) typically requires microbiological persistence (ie, multiple positives) and longitudinal clinical follow-up. Of note, for extrapulmonary infection, isolation of *M. abscessus* from a single, typically sterile site, body fluid, or tissue specimen is usually sufficient to meet diagnostic criteria.

## IS IT IMPORTANT TO REQUEST SUBSPECIES IDENTIFICATION?

Although some laboratories still report *M. abscessus* isolates as part of an *M. abscessus/chelonae* complex, increasing use of molecular methods such as *rpoB* gene sequencing should be able to distinguish between the 2 organisms and the subspecies that comprise the *M. abscessus* group: *abscessus*, *massiliense*, and *bolletii* [8, 13]. Although virulence appears to be similar, the distinction between subspecies is important clinically. The majority of *M. abscessus* subsp. *abscessus* and *M. abscessus* subsp. *bolletii* isolates have intrinsic macrolide resistance due to the presence of a functional inducible erythromycin ribosomal methylase (*erm*) 41 gene [11]. In contrast, this gene is present but not functional in most *M. abscessus* subsp. *massiliense* isolates, which are typically susceptible to macrolides, conferring a better treatment prognosis [14]. There is growing interest in the use of whole-genome sequencing (WGS) to determine subspecies identification, although further optimization of this tool is needed before it is ready for clinical use [15].

## HOW SHOULD DRUG SUSCEPTIBILITY TEST RESULTS BE INTERPRETED?

The decision to request drug susceptibility testing (DST) should be based on the likelihood that the isolate recovered is of clinical significance, based on the clinical and microbiologic criteria described earlier. Although published data on the exact role of DST and its potential to impact or predict outcomes with *M. abscessus* are limited, most experts suggest performing DST for clinically significant *M. abscessus* isolates [12, 16]. Broth microdilution has been recommended by the Clinical and Laboratory Standards Institute (CLSI) for RGM DST [17]. Nonetheless, it should be noted that there is limited clinical validation of proposed minimum inhibitory concentrations (MICs) to define susceptibility for most antibiotics for *M. abscessus* [12]. Recognizing limitations of proposed MIC thresholds, macrolides are the only commonly used oral antibiotics with reliable in vitro susceptibility against *M. abscessus* (in the absence of inducible macrolide resistance), although the less common antibiotics clofazimine and bedaquiline usually have in vitro susceptibility as well. Tigecycline is the most likely intravenous antibiotic to demonstrate in vitro susceptibility, followed by amikacin (which typically has the lowest MIC of the aminoglycosides) and imipenem or cefoxitin [8, 18]. Of note, clarithromycin results are generally predictive of the MICs for azithromycin [17].

Screening for inducible macrolide resistance by determining the presence and functional status of the *erm*41 gene is strongly recommended. This is now typically done by 16S rRNA gene sequence analysis rather than a 14-day prolonged incubation [19], although there are situations where genotypic and phenotypic discordance occur due to mutations in other genes such as *rrl* [20]. Clinicians reliant on testing from commercial laboratories should be aware that only a few laboratories screen for inducible macrolide resistance, so the authors recommend the use of laboratories with experience in performing mycobacterial DST.

Various studies have demonstrated that patients with clarithromycin-susceptible *M. abscessus* strains are more likely to have successful treatment outcomes [21, 22]. Aside from macrolide susceptibility, there is a limited body of evidence correlating DST results with clinical outcomes. Nonetheless, phenotypic DST should inform treatment decisions, particularly when available options are limited. For example, despite the appearance of in vitro phenotypic resistance, many experts may consider inclusion of antibiotics with MICs that are close to the dichotomous thresholds used to define resistance vs susceptibility.

## WHAT IS THE OVERALL APPROACH TO THERAPY AND PROGNOSIS?

There are limited controlled and/or randomized data for treatment of *M. abscessus* lung disease. Consequently, small studies and expert guidance play a large role in recommendations, resulting in heterogeneity in practice [8, 23]. Unlike other NTM such as *M. avium* complex, there is no antibiotic regimen that has been demonstrated to result in sustained long-term sputum culture conversion in patients with pulmonary *M. abscessus* disease [8]. When embarking on a therapeutic course, the goals of therapy should be considered, along with treatment response and toxicity. In instances of chronic immunosuppression or extensive pulmonary disease, cure (defined as 12 months of negative sputum cultures) may be an unrealistic outcome compared with a long-term goal of clinical control. Recognizing the increasing rates of colonization and pulmonary disease, specific guidelines and management strategies have also been developed for patients with CF [12]. To mitigate the development of drug resistance, clinicians caring for CF patients colonized with *M. abscessus* should try to avoid the use of antibiotics such as macrolides, aminoglycosides, imipenem, and linezolid to treat other infections that may occur in this population [12]. *M. abscessus* pulmonary disease in patients with CF is considered to be a contraindication to lung transplantation by some centers, although there are increasing data demonstrating good outcomes in this patient population when an aggressive management and surveillance strategy is used [7, 24].

The duration and intensity of *M. abscessus* therapy are often individualized to microbiological, radiographic, and clinical progress. Given the difficulty of achieving cure, symptom and radiographic improvement are the most useful markers to guide treatment duration and determine success for pulmonary *M. abscessus* [8], although in clinical practice, duration of therapy is frequently limited by antibiotic toxicity.

The following approach (also, see Table 1) is generally recommended with drugs considered susceptible based on DST [8]. An induction phase usually includes 1 or more intravenous agents for at least 8 weeks depending on tolerability. Subsequently, a consolidation phase with oral or inhaled agents is usually undertaken for 12–18 months, with consideration for the suppressive phase in patients at high risk of relapse [7]. However, the ultimate regimen and duration depend on the extent of drug resistance and tolerability of therapy. In some instances of drug toxicity, periods of treatment are sometimes punctuated by medication holidays, particularly with consideration of overall treatment goals. For macrolide-susceptible isolates (ie, *massiliense*), an oral macrolide should be part of the regimen in all phases. For macrolide-resistant isolates (ie, *abscessus*), there may still be a role for macrolide inclusion, particularly when other treatment options based on DST are limited or not tolerated, although this remains a matter of debate [25]. Azithromycin is typically the favored macrolide, given in vitro data to suggest that azithromycin is less likely to induce *erm* gene resistance compared with clarithromycin [25] and clinical data demonstrating better treatment outcomes (sustained culture conversion) with azithromycin [22]. Table 1 suggests potential regimens for each phase of therapy, although these will vary according to the isolate DST.

**Table 1.** Approach to Treatment of Pulmonary *M. abscessus* Disease

Phase of Treatment	No. and Type of Antibiotics	Suggested Regimens^a^
Induction (up to 8 wk or longer depending on extent of disease, resistance pattern, underlying host)	3–4 antibiotic regimen, at least 1–2 active IV agents	IV amikacin + IV imipenem or cefoxitin or tigecycline + azithromycin + clofazimine
Consolidation (12–18 mo)	2–3 active^b^ oral or inhaled antibiotics	Azithromycin^b^ + clofazimine + 3rd agent (alterative oral such as linezolid^c^ or bedaquiline if susceptibilities allow after expert consultation, particularly for salvage therapy, otherwise inhaled amikacin^d^)
		OR
		In individuals with limited oral options, prolonged induction phase for as long as tolerable, often followed by medication holiday of variable duration
Suppressive (consideration in some patients)	2 active^b^ oral/inhaled antibiotics	Azithromycin^b^ +
		Clofazimine +/-
		Inhaled amikacin^d^
		OR
		In individuals with limited oral options, cessation of therapy after prolonged IV induction with clinical monitoring and potential future IV therapy if worsening

Abbreviation: IV, intravenous.

^a^This will always depend on the susceptibility profile, including assessment for a functional *erm* gene.

^b^Azithromycin should not be considered one of the fully active antibiotics in an isolate with likely/known inducible macrolide resistance.

^c^Linezolid dose is typically 600 mg daily, and concomitant vitamin B6 is recommended by the authors.

^d^Despite limited data on treatment outcomes with inhaled amikacin, this is often used as a third agent in clinical practice, although it is not typically assumed to be a fully active drug. Studies evaluating liposomal inhaled amikacin (Arikayce) demonstrate poorer outcomes in patients with *M. abscessus* infections than for those with *M. avium* complex infections (although numbers were small), so this agent is not being pursued for these infections.

Management of pulmonary *M. abscessus* disease is difficult, and treatment outcomes are generally poor [4, 8]. In the absence of surgical resection, this infection is often deemed incurable. Studies evaluating *M. abscessus* outcomes have reported treatment success rates of 30%–50% for subspecies *abscessus* vs 80%–90% for *massiliense*, likely due to the ability to use macrolide-based therapy [4, 21, 22]. Of note, in the absence of macrolide susceptibility, suppressive therapy is often not possible, and a strategy of induction treatment with a duration limited by toxicity, followed by periods of medication holidays, is the more common clinical pathway.

## IS INTRAVENOUS THERAPY NECESSARY?

Given paucity of nonintravenous (non-IV) options, IV therapy is needed for at least the beginning of almost all treatment courses. As intravenous amikacin has the most appealing time-kill curves of the frequently susceptible agents and a favorable dosing schedule compared with other parenteral agents, it should be included in most regimens using 3 times/wk dosing [8, 26, 27]. Ideally IV amikacin would be used for no longer than 8–12 weeks to reduce toxicity risk, with monitoring for the development of nephro- or ototoxicity [8, 23, 28]. However, in patients with limited consolidation options or extensive disease, IV therapy may be extended for as long as tolerable. Generally, an IV beta-lactam (imipenem or cefoxitin are most likely to demonstrate acceptable MICs [29]) should be added to IV amikacin for the initial phase of therapy. There is evidence demonstrating that dual beta-lactam therapy exhibits synergy against *M. abscessus* [8, 30–32], although clinical experience is limited, and overlapping toxicities should be considered. Tigecycline also typically has reliable in vitro activity [18]. Formal clinical data are limited, and toxicity may be treatment-limiting at the traditional 50-mg IV q12 dosing, yet there is increasing experience with doses as low as 25–50 mg IV q24 [18]. Successful strategies to improve tolerability include slow escalation of dosing and premedication with anti-emetics [33]. This agent may be used initially with IV amikacin and an IV beta-lactam in patients with extensive/severe or disseminated disease, or as an alternative to IV amikacin or IV beta-lactams if these cannot be used [8, 26, 33].

## WHAT ABOUT REPURPOSED AND NEW DRUGS SUCH AS CLOFAZIMINE AND BEDAQUILINE?

Clofazimine is often the only oral antibiotic, other than macrolides, with favorable susceptibilities, and small case series suggest it could be a useful agent [34, 35]. Alternative oral antibiotic options that have more variable susceptibility patterns and limited clinical data include the fluoroquinolones (moxifloxacin is favored if DST from a reputable reference laboratory suggests activity), oxazolidinones (linezolid and tedizolid), and bedaquiline [36–39]. In a setting of favorable susceptibilities or salvage therapy, any of these agents could be considered as part of the treatment regimen. Inhaled amikacin is typically only used as an added agent in a suppressive regimen or as salvage therapy, where it *may* improve treatment responses in patients with refractory NTM disease, including *M. abscessus* [40]. However, other data on liposomal inhaled amikacin (recently approved for refractory pulmonary MAC disease) suggest a lack of benefit in patients with *M. abscessus*, albeit based on small numbers of patients [41].

## WHAT IS THE ROLE FOR AIRWAY CLEARANCE STRATEGIES AND SURGERY?

Evidence for the efficacy of airway clearance strategies in pulmonary NTM infection is limited. As exacerbations of bronchiectasis often complicate the assessment and management of these patients, strategies aimed at bronchiectasis per se, such as airway clearance, are often recommended as an adjunctive management strategy. Observational data suggest that chest physical therapy may improve symptoms, even in the absence of antimicrobial therapy, and for the majority of individuals who have underlying bronchiectasis, this is a strong recommendation [42, 43]. There is stronger evidence to support the use of pulmonary rehabilitation in those with bronchiectasis [43].

Observational cohort data suggest that rates of culture conversion are higher in patients with pulmonary *M. abscessus* who receive surgery in addition to antibiotic therapy, although specific surgical criteria have not been established [26, 44, 45]. Surgery is typically recommended in 3 situations: (a) failure of medical therapy, particularly in the setting of macrolide or other significant drug resistance; (b) management of symptoms including refractory hemoptysis; and, in limited cases, (c) to limit or slow down the progression of disease in scenarios where the goal for surgery is to stabilize rather than eradicate infection, for example, by debulking the worst areas of parenchymal damage to limit spillage of secretion into previously healthy areas [46]. This type of surgery should be performed in centers with expertise in the medical and surgical management of NTM disease, and it is recommended that patients being considered for surgery have focal parenchymal disease amenable to resection, have adequate cardiopulmonary reserve, and have an optimized nutritional status [8]. Where possible, a minimally invasive approach and use of anatomic lung resection may result in improved clinical outcomes at experienced centers [46, 47]. Many questions remain regarding the optimal timing for surgery, including duration of pre- and postoperative antibiotic therapy, which would be better informed by well-designed clinical trials. Experts recommend antimycobacterial pretreatment before resection to potentially decrease bacillary burden, with the goal of reducing postoperative complications including bronchopleural fistula formation, followed by a prolonged postoperative treatment course [7, 26].

## CONCLUSIONS

Diagnosis of *M. abscessus* disease is improving with the use of molecular tools, although subspecies identification is not often available to clinicians. A composite assessment (clinical, radiographic, microbiologic) is still the mainstay of determining whether a patient has true disease that warrants intervention. Treatment is complicated by the need for multiple oral and parenteral antibiotics, medication-related toxicities, and the lack of robust comparative clinical outcome data to guide decisions about choice of therapy and adjunctive measures such as surgical resection. Nonetheless, the aggressive and systematic approach we have outlined could help to guide clinicians regarding the management of patients with this complex disease.
